# Can the cytokine adsorber CytoSorb^®^ help to mitigate cytokine storm and reduce mortality in critically ill patients? A propensity score matching analysis

**DOI:** 10.1186/s13613-021-00905-6

**Published:** 2021-07-22

**Authors:** Christina Scharf, Ines Schroeder, Michael Paal, Martin Winkels, Michael Irlbeck, Michael Zoller, Uwe Liebchen

**Affiliations:** 1 Department of Anesthesiology, University Hospital LMU Munich, Marchioninistrasse 15, 81377 Munich, Germany; 2 Institute of Laboratory Medicine, University Hospital LMU Munich, Munich, Germany

**Keywords:** Cytosorb^®^, Cytokine storm, Interleukin-6, Mortality, Propensity score matching

## Abstract

**Background:**

A cytokine storm is life threatening for critically ill patients and is mainly caused by sepsis or severe trauma. In combination with supportive therapy, the cytokine adsorber Cytosorb^®^ (CS) is increasingly used for the treatment of cytokine storm. However, it is questionable whether its use is actually beneficial in these patients.

**Methods:**

Patients with an interleukin-6 (IL-6) > 10,000 pg/ml were retrospectively included between October 2014 and May 2020 and were divided into two groups (group 1: CS therapy; group 2: no CS therapy). Inclusion criteria were a regularly measured IL-6 and, for patients allocated to group 1, CS therapy for at least 90 min. A propensity score (PS) matching analysis with significant baseline differences as predictors (Simplified Acute Physiology Score (SAPS) II, extracorporeal membrane oxygenation, renal replacement therapy, IL-6, lactate and norepinephrine demand) was performed to compare both groups (adjustment tolerance: < 0.05; standardization tolerance: < 10%). *U*-test and Fisher’s-test were used for independent variables and the Wilcoxon test was used for dependent variables.

**Results:**

In total, 143 patients were included in the initial evaluation (group 1: 38; group 2: 105). Nineteen comparable pairings could be formed (mean initial IL-6: 58,385 vs. 59,812 pg/ml; mean SAPS II: 77 vs. 75). There was a significant reduction in IL-6 in patients with (*p* < 0.001) and without CS treatment (*p* = 0.005). However, there was no significant difference (*p* = 0.708) in the median relative reduction in both groups (89% vs. 80%). Furthermore, there was no significant difference in the relative change in C-reactive protein, lactate, or norepinephrine demand in either group and the in-hospital mortality was similar between groups (73.7%).

**Conclusion:**

Our study showed no difference in IL-6 reduction, hemodynamic stabilization, or mortality in patients with Cytosorb^®^ treatment compared to a matched patient population.

## Introduction

Although there is no uniform definite clinical picture of a “cytokine storm”, it involves a massive release of cytokines into the bloodstream [1]. A wide variety of etiologies can trigger cytokine storm, the most common being sepsis, severe trauma, liver failure, and CART-T cell therapy [2, 3]. All origins lead to cytokine-mediated vasodilation and capillary leakage, which can ultimately cause circulatory insufficiency [4]. The underlying pathology should be eliminated as soon as possible in order to interrupt the release of cytokines.

In the case of septic shock, the infection leads to an activation of the immune system and results in the release of cytokines [5]. Causal therapeutic options include source control (if present) and the prompt administration of effective anti-infective drugs [6, 7].

In addition to causal therapy, supportive therapeutic strategies play an important role, especially in the initial phase of the disease. Forced volume therapy and the administration of hydrocortisone can therefore be helpful to stabilize patients’ hemodynamics [8]. An extended supportive therapeutic intervention is the use of the cytokine adsorber Cytosorb^®^ (CS). It is usually integrated into an existing renal replacement procedure and promises the adsorption of molecules with a size of approximately 5–55 kDa [9]. Because acute kidney injury (AKI) with the need of kidney replacement therapy (KRT) frequently occurs in those patients [10], integration can be achieved easily in this respect.

Interleukin-6 (IL-6) is a cytokine that is measured as a matter of clinical routine in a lot of hospitals, whereby its concentration might be a predictor of patient´ mortality [11]. The half-life of the majority of cytokines is very short, ranging from 10 to 20 min, so a rapid decrease can be expected if the causative reason is eliminated. Therefore, the decrease in IL-6 can also be used as a predictor of therapeutic success [12].

As most cytokines have a molecular mass of approximately 25 kDa (in a range of 6–70 kDa), elimination by CS, in contrast to high-flux dialysis membranes, is technically possible. This was also demonstrated by in vitro data from Harm et al., which showed that various cytokines were eliminated from human plasma using CS [13].

Different authors showed a decrease in cytokines in patients treated with CS [14, 15]; however, it remains unclear whether a comparable decrease would also have been observed without the use of CS, since control groups were missing. A randomized controlled trial recently published by Supady et al. showed no difference in IL-6 reduction with and without CS in patients infected with Sars-CoV-2 and supported by extracorporeal membrane oxygenation [16]. In addition, the real adsorption capacity and saturation of CS is not yet clear. It must also be taken into account that ultimately not only cytokines are selectively adsorbed, but also other substances with the appropriate size such as myoglobin [9]. This lack of saturation kinetics makes the evaluation of performance and efficacy enormously difficult. Brouwer et al. showed that the use of CS could lead to a lower mortality than expected as compared to a control group in a retrospective analysis [17]. However, the patient matching procedure was criticized [18].

In summary, robust data investigating the specific effects of CS are very sparse and prospective trials are rarely available. To start closing this gap, a propensity score (PS) matching analysis was investigated to compare patients with cytokine storm (IL-6 > 10,000 pg/ml) treated with standard therapy and with standard therapy supported by CS.

Points of interest were as follows: (i) whether the use of CS led to a faster reduction in IL-6 compared to standard therapy without the use of CS; (ii) whether there was a faster hemodynamic stabilization with CS therapy; (iii) and whether mortality was lower in patients treated with CS than those without CS treatment.

## Methods

### Study setting

This was a monocentric, PS matching study investigating the effect of CS therapy on critically ill patients with cytokine storm. Patients were included between October 2014 and May 2020 during their stay at the ICU at LMU hospital. The local institutional review board approved the study (Registration Number 20-477).

### Laboratory measurements and data collection

All clinical-chemical parameters were determined with standard clinical chemistry tests. The technique of IL-6 measurement was consistent during the study period. It was quantified with the Elecsys IL-6 chemiluminescence immunoassay on the standard clinical chemistry analyzer Cobas 8000 from Roche Diagnostics (Mannheim, Germany). For data evaluation, demographic data, clinical variables, and laboratory variables were collected from the laboratory and patient information system.

### Study population

All patients with an interleukin-6 (IL-6) > 10,000 pg/ml were screened for inclusion in the study. Inclusion criterion was a regular (at least twice daily) measurement of IL-6. Patients were divided into two groups: with CS therapy and without CS therapy. Group 1 included patients that received CS therapy for at least 90 min. For each patient, only the first treatment cycle that met the inclusion criteria was included. Group 2 was defined as IL-6 > 10,000 pg/ml without subsequent CS treatment.

### Blood sampling

In the data evaluation, three time points were considered depending on CS treatment (group 1) or on IL-6 > 10,000 pg/ml (group 2):

- d-1: 12–24 h before d0;
- d0: 0–12 h before starting CS therapy (group 1) or the measured IL-6 > 10,000 pg/ml (group 2);
- d1: 12–24 h after starting CS therapy (group 1) or 12–24 h after d0 IL-6 (group 2).

### Statistical analysis

A statistical analysis was performed with IBM SPSS statistics (Version 26.0. IBM Corp., Armonk, NY, USA). The effect of CS treatment on the reduction in IL-6, C-reactive protein (CRP), lactate, and norepinephrine was investigated using the Wilcoxon test with associated samples due to the lack a normal distribution of parameters. The relative change was calculated with: 100 – [(100/parameter d1) × parameter d0]. Differences in baseline parameters in both groups were detected with the Mann–Whitney *U-*test. A PS matching (1:1) was performed to compare both groups. Simplified Acute Physiology Score (SAPS) II, IL-6 d0, renal replacement therapy, extracorporeal membrane oxygenation, lactate concentration d0, and norepinephrine requirement d0 were used as predictors. The adjustment tolerance was < 0.05 and the nearest neighbor method was used. The standardized difference “*d*” [*d* = (mean A − mean B)/pooled standard deviation of both groups] should be < 10% after matching as a quality criterion [19]. Finally, the relative changes in IL-6, CRP, lactate, norepinephrine demand, and in-hospital mortality were investigated in the matched groups using the *U*-test.

## Results

### Demographic and clinical data

In total, 143 patients were included in the evaluation (group 1: 38; group 2: 105). The underlying diseases resulting in admission to the ICU in patients allocated to group 1 were as follows: septic shock (47.4%), acute respiratory distress syndrome (ARDS, 36.8%), polytrauma (7.9%), and others (7.9%). The median SAPS II score in those patients was high at 80 points. Furthermore, IL-6 and CRP levels before CS therapy were also very high with a median of 60,529 pg/ml and 14.9 mg/dl, respectively. The mean relative reduction of IL-6 from d0 to d1 was 77.2%.

The underlying diseases resulting in admission to the ICU in patients allocated to group 2 were as follows: sepsis (different reasons except urosepsis) (21.0%), urosepsis (15.2%), septic shock (15.2%), ARDS (13.3%), hemorrhagic shock (8.6%), pneumonia (6.7%), polytrauma (4.8%), and others (15.2%). These patients had a lower SAPS II score (62 points) and less need for organ replacement procedures. Furthermore, the IL-6 at d0 was substantially lower at 25,660 pg/ml. The mean relative reduction of IL-6 from d0 to d1 was 89.0%.

### Change in IL-6, CRP, lactate and norepinephrine demand with and without Cytosorb^®^ before PS matching

There was a significant (*p* < 0.001) decrease in IL-6 during CS therapy (median relative reduction: 79.1%). Furthermore, there was a significant (*p* = 0.035) reduction in norepinephrine demand (median relative reduction: 27.7%). In contrast, there was no significant change in CRP (*p* = 0.965) or lactate (*p* = 0.455).

There was also a significant (*p* < 0.001 each parameter) decrease in IL-6, lactate, and norepinephrine demand in patients without CS therapy. Furthermore, there was a significant (*p* < 0.001) increase in CRP in the same period.

### Comparison of baseline characteristics in the two groups

*U*-test was used for the detection of baseline differences at d0. Patients allocated to group 1 (CS therapy) needed ECMO therapy and KRT more often. Moreover, SAPS II and in-hospital mortality were higher than in patients allocated to group 2. Furthermore, IL-6, lactate, and norepinephrine demand were significantly higher in patients allocated to group 1. No significant difference was seen for age, gender, BMI, 48-h mortality, or CRP. Detailed statistical results are presented in Table 1.

**Table 1:** Patient characteristics and laboratory measurements in both groups before matching

	Group 1: n (%) or median [range: min, max]	Group 2: n (%) or median [range: min, max]	*p*-value (*U*-test)
Number	38 (100)	105 (100)	
Patient characteristics			
Age (years)	56 [19, 88]	61 [17, 91]	0.080
Male/female	28 (73.7)/10 (26.3)	59 (56.2)/46 (43.8)	0.059
BMI (kg/m^2^)	26.1 [16.1, 50.2]	24.8 [13.1, 42.9]	0.260
ECMO therapy	13 (34.2)	14 (13.3)	0.005*
KRT	38 (100)	34 (32.4)	< 0.001*
48 h mortality	4 (28.9)	11 (19.0)	0.993
In-hospital mortality	25 (65.8)	46 (43.8)	0.01*
SAPS II d0	80 [38, 118]	62 [27, 107]	< 0.001*
Laboratory measurements			
IL-6 d-1 (pg/ml)	7520 [21, 1,700,000]	206 [22, 9377]	
IL-6 d0 (pg/ml)	60,529 [10,108, 84,000,000]	25,660 [10,051, 600,000]	0.002*
IL-6 d1 (pg/ml)	13,791 [265, 500,000]	2826 [31, 260,000]	
CRP d-1 (mg/dl)	9.8 [0.1, 31.9]	12.8 [0.1, 37.8]	
CRP d0 (mg/dl)	14.9 [0.3, 47.1]	11.1 [0.1, 46.9]	0.239
CRP d1 (mg/dl)	15.0 [0.7, 37.9]	21.6 [1.1, 56.2]	
Lactate d-1 (mmol/l)	6.2 [0.6, 13.2]	1.5 [0.5, 15.0]	
Lactate d0 (mmol/l)	9.0 [1.0, 24.0]	4.0 [0.8, 26.0]	< 0.001*
Lactate d1 (mmol/l)	7.7 [1.2, 25.8]	3.0 [0.7, 21.0]	
Total bilirubin d0 (mg/dl)	2.1 [0.5, 23.6]	1.7 [0.3, 25.5]	0.176
Norepinephrine d-1 (mg/h)	1.6 [0.0, 7.0]	0.5 [0.0, 10.0]	
Norepinephrine d0 (mg/h)	3.8 [0.9, 10.0]	2.0 [0.0, 16.0]	< 0.001*
Norepinephrine d1 (mg/h)	2.2 [0.4, 20.0]	1.0 [0.1, 10.0]	

d0: 0–12 h before starting CS therapy (group 1) or the measured IL-6 > 10,000 pg/ml (group 2), d1: 12–24 h after starting CS therapy (group 1) or 12–24 h after d0 IL-6 (group 2)

*BMI* body mass index, *ECMO* extracorporeal membrane oxygenation, *ICU* intensive care unit, *SAPS* Simplified Acute Physiology Score, *CRP* C-reactive protein, * *p* < 0.05

### Propensity score matching with baseline differences

ECMO therapy, KRT, SAPS II d0, IL-6 d0, lactate d0, and norepinephrine demand d0 were significantly different in the two groups. Consequently, a PS matching analysis was conducted using the above-mentioned parameters as predictors. Nineteen pairs were successfully matched according to the defined matching criteria. Patient characteristics of the matched patient population can be found in Table 2. There was no longer any difference in the baseline parameters, and the mean difference from the mean was less than 5.9% for all parameters.

**Table 2:** Patient characteristics and laboratory measurements after PS matching analysis

	Group 1: n (%) or mean [range: min, max]	Group 2: n (%) or mean [range: min, max]	*p*-value (*U*-test/Fisher test)	Difference from mean (%)
Number	19 (100)	19 (100)		
Patient characteristics				
ECMO therapy	4 (21.0)	4 (21.0)	1.00	0.0
KRT	19 (100)	19 (100)	1.00	0.0
SAPS II d0	77 [38, 118]	75 [48, 100]	0.644	1.7
48-h mortality	1 (5.3)	3 (15.8)	0.583	
In-hospital mortality	14 (73.7)	14 (73.7)	1.00	
Surgical intervention	9 (47.4)	8 (42.1)	0.79	
Laboratory measurements				
IL-6 d0 (pg/ml)	58,385 [10,108, 206,000]	59,812 [10,051, 600,000]	0.181	1.2
IL-6 d1 (pg/ml)	16,314 [461, 123,000]	27,445 [30, 260,000]		
CRP d0 (mg/dl)	14.5 [0.3, 47.1]	16.3 [0.1, 46.9]	0.817	5.8
CRP d1 (mg/dl)	13.8 [0.7, 28.1]	20.9 [1.1, 41.6]		
Lactate d0 (mmol/l)	7.8 [1.0, 17.6]	8.2 [0.8, 26.0]	0.729	2.5
Lactate d1 (mmol/l)	8.6 [1.2, 17]	6.7 [0.9, 18]		
Nor d0 (mg/h)	3.8 [0.9, 8.0]	3.6 [0.0, 16.0]	0.223	2.7
Nor d1 (mg/h)	2.8 [0.4, 11.5]	2.3 [0.3, 6.0]		

d0: 0–12 h before starting CS therapy (group 1) or the measured IL-6 > 10,000 pg/ml (group 2), d1: 12–24 h after starting CS therapy (group 1) or 12–24 h after d0 IL-6 (group 2)

*BMI* body mass index, *ECMO* extracorporeal membrane oxygenation, *ICU* intensive care unit, *SAPS* Simplified Acute Physiology Score, *CRP* C-reactive protein, *Nor* norepinephrine

The reasons for the admission to the ICU were as follows: sepsis or septic shock (50.0%), ARDS (18.4%), polytrauma (13.2%), abdominal emergency (7.9%), solid organ transplantation (5.3%), and others (5.3%). The different reasons resulting in cytokine storm in patients allocated to group 1 were as follows in descending order: septic shock (36.8%), ischemia (21.1%), ARDS (15.8%), anastomotic insufficiency (10.5%), polytrauma (10.5%), and unclear cause (5.3%). The different reasons for patients allocated to group 2 were as follows: septic shock (42.1%), ischemia (21.1%), ARDS (15.8%), anastomotic insufficiency (10.5%), polytrauma (5.3%), and unclear cause (5.3%). There was no relevant difference in the origin of the cytokine storm in both groups. Nine and eight patients allocated to group 1 and 2, respectively, had a surgical intervention (from 48 h prior until 48 h after study period).

The median delay from ICU admission to CS (group 1) or to IL-6 > 10,000 pg/ml (group 2) was 0 days in both groups (IQR group 1: 0, 2.5 days, IQR group 2: 0, 0.75 days). The median treatment with CS until d1 was 9 h (range: 7–12 h). There was no change of the cartridge in the study period.

### Comparison of the matched study populations

**Fig. 1:**
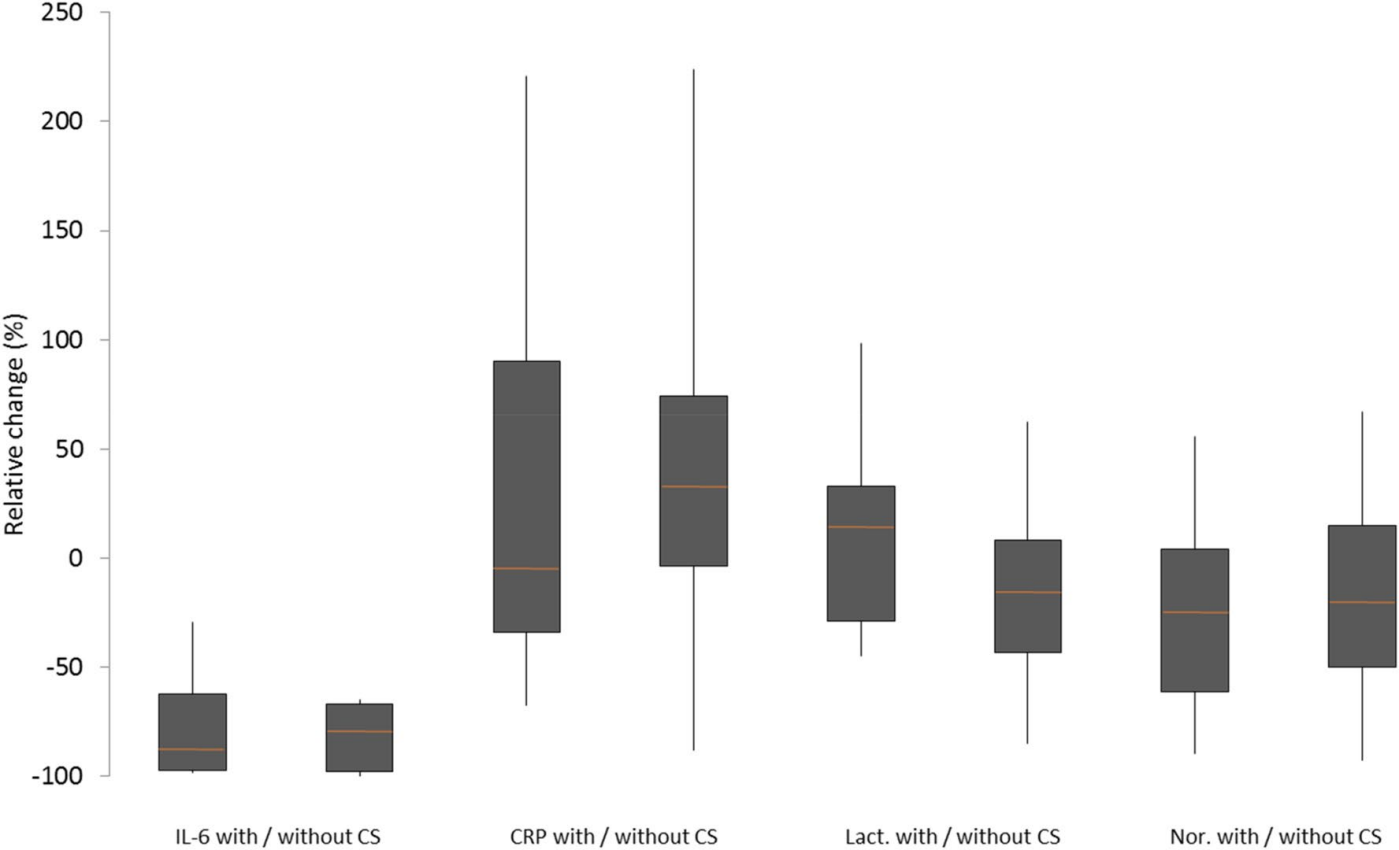
Relative change of IL-6, CRP, lactate and norepinephrine in patients with and without Cytosorb^®^ therapy in the matched population. *IL-6* interleukin-6, *CRP* C-reactive protein, *Lact* lactate, *Nor* norepinephrine demand, *CS* Cytosorb^®^

**Fig. 2:**
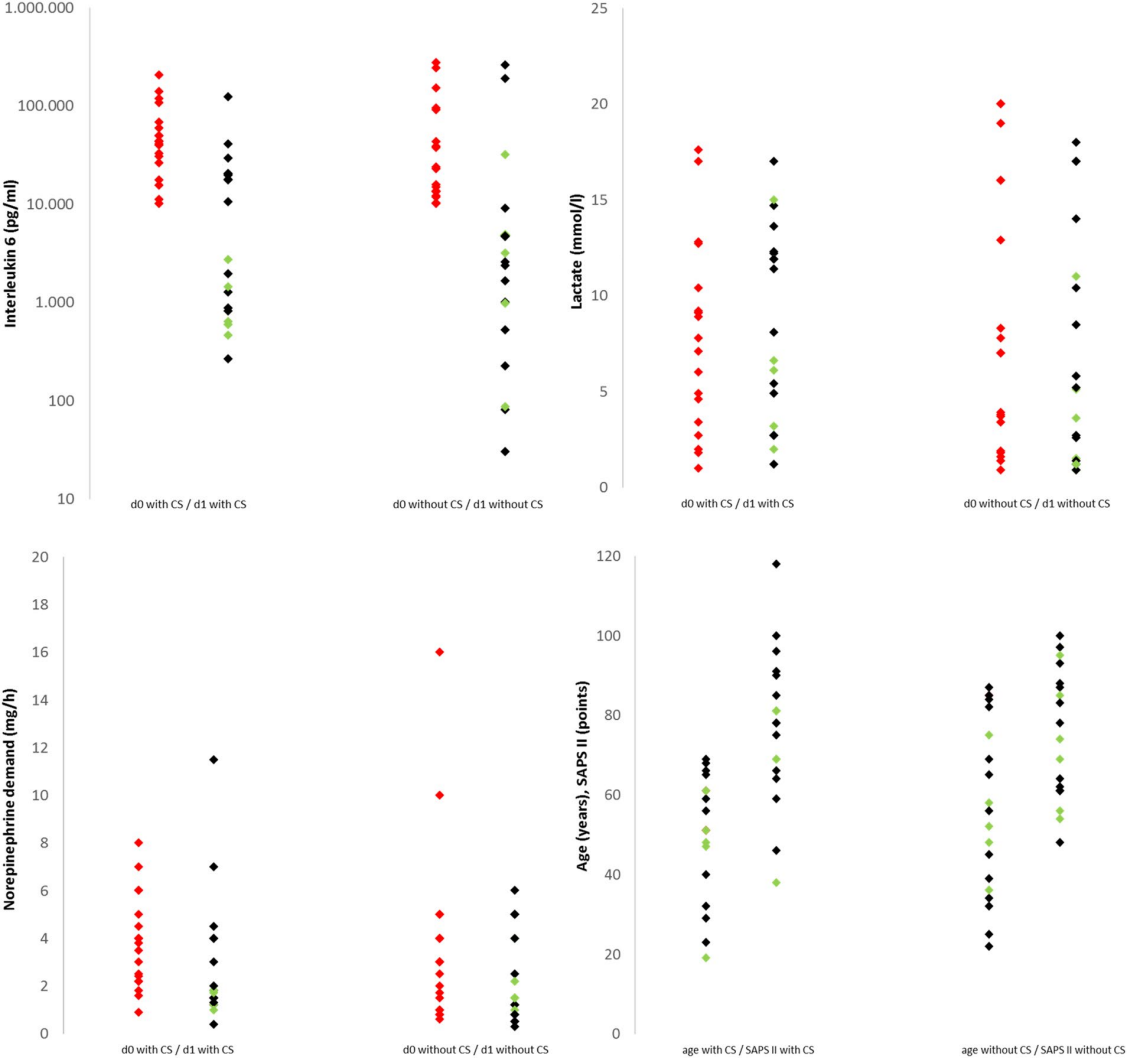
Interleukin-6, lactate and norepinephrine demand on day 0 and day 1, and SAPS II and age on d0 in patients with and without CytoSorb^®^ therapy. green dots, patient survived; black dot, patient died in the hospital; CS, CytoSorb^®^; SAPS, Simplified Acute Physiology Score; CRP, C-reactive protein; d0, 0–12 h before starting CS therapy (group 1) or the measured IL-6 > 10,000 pg/ml (group 2); d1, 12–24 h after starting CS therapy (group 1) or 12–24 h after d0 IL-6 (group 2); Nor, norepinephrine

There was a significant decrease in IL-6 in patients with (*p* < 0.001) and without CS therapy (*p* = 0.005). The median relative reduction with and without CS was 89% and 80%, respectively. There was no significant difference in the relative reduction (*p* = 0.708) between the two groups. However, there was no significant change in CRP in patients with CS (*p* = 0.936), i.e., it tended to increase without CS treatment (*p* = 0.058). While lactate slightly increased with CS, it tended to decrease without CS. Thus, there was an almost significant difference (*p* = 0.057) in the relative change in lactate (CS: median increase of 15%, without CS: median decrease of 15%). There was no significant change in norepinephrine demand in both groups and no significant difference in the 48-h mortality (*p* = 0.583) and in-hospital mortality (*p* = 1.00) between the groups. Detailed changes in IL-6, CRP, lactate, and norepinephrine demand in both groups can be found in Fig. 1. Furthermore, IL-6, lactate and norepinephrine demand for d0 and d1, and age and SAPS II for d0 are displayed in Fig. 2 for both groups.

## Discussion

The principle of cytokine adsorption is a promising technique to mitigate cytokine storm in critically ill patients [20]. However, there are only limited prospective data with control groups available that investigate the elimination of cytokines using CS [21–23]. Although a decrease in cytokines was described by different authors [15, 24], this could be due to causal therapy approaches such as anti-infective or supportive therapy [25].

Our primary dataset before PS matching suggested a rapid and significant decrease in IL-6 12–24 h after the initiation of CS therapy (median: 60,529 pg/dl → 13,791 pg/dl, mean relative reduction: 77%), which is in agreement with previous data. However, a significant and comparable decrease was also observed in patients without CS treatment (median: 25,660 pg/dl → 2826 pg/dl, mean relative reduction 89%). Furthermore, a reduced norepinephrine demand can be seen in both groups after 12–24 h. Since patients with and without CS differed significantly in terms of disease severity and baseline parameters, the groups cannot be directly compared. Therefore, PS matching was performed to enable the retrospectively collected groups to be compared [26]. All variables that differed significantly in the two groups were utilized as predictors in the matching analysis. Quality control of the matched patient groups (relative difference from mean) showed a deviation of < 5.9% in all variables, indicating no imbalance between the groups [27]. Retrospectively, no reason could be identified as to why group 2 did not receive CS. This was at the discretion of the responsible physicians. It should be noted in this context that the availability of therapy would always have been available in both groups (established KRT, CS being available at all times throughout the study period). Moreover, the propensity score was never zero or one, which is a prerequisite of the positivity assumption [28]. There is a commitment to discuss the use of CS in patients with KRT and IL-6 > 10,000 pg/ml among all treating physicians, so that the therapy was considered in every patient. Furthermore, there were no relevant differences in the causes of cytokine storm in both groups and the duration of CS treatment was comparable.

After PS matching, there was a significant decrease in IL-6 in patients treated with CS (mean: 58,385 pg/ml → 16,314 pg/ml). However, a significant decrease was also observed in patients that did not receive CS treatment (mean: 59,812 pg/ml → 27,445 pg/ml). A comparison of the decrease (relative reduction) showed no significant difference. This finding is consistent with one of the few available prospective randomized trials, wherein no difference in IL-6 progression was detectable [29]. It should be noted, of course, that CS might eliminate other cytokines [30, 31], and these might accumulate without CS. These cytokines, such as IL-8 and IL-10, were not measured in the present study. Therefore, a statement about their elimination cannot be made. Additionally, it remains unclear how quickly cartridge saturation occurs at enormously high IL-6 levels. There is no definitive information on this either in the literature or from the company. A prospective study that calculates the real elimination performance by measuring the cytokines before and after the cartridge would be relevant in this respect.

The previously published literature indicated a hemodynamic stabilization in patients during CS treatment [14, 32]; however, these analyses lacked a control group. We also detected hemodynamic stabilization in the form of a reduced norepinephrine demand in patients treated with CS. However, there was also a comparable reduction in the control group. It is worth mentioning that the lactate levels tended to increase with CS and tended to decrease without CS, which rather indicates an advantage in the absence of CS. That the use of CS can also be disadvantageous was shown by a recently published randomized trial, which detected a significantly higher mortality in the CS group [16]. Furthermore, Poli et al. also failed to find any perioperative advantage in cardiac surgery patients with regard to hemodynamics in a randomized, prospective study with and without CS treatment [33].

Our data showed that there was an equal mortality rate and therefore no survival advantage in one of the two groups (mortality rate: 73.7%). Several studies observed a lower mortality rate than predicted based on intensive care scores [14, 17]. We believe that these results should be viewed with caution. With modern intensive care medicine, scores slightly overestimate mortality rates [34], which can also be seen in our population (the predicted mortality in both groups was ~ 90%). In the context of multifactorial therapy, it is difficult to attribute a survival benefit to a single device, as Brouwer et al. did [35]. To do this would require a larger patient population.

## Limitations

Our study has a few limitations. Even if there was no difference in hemodynamic stabilization and mortality in a matched patient population, it is important to note that PS matching cannot replace a prospective randomized trial. Because statistical power is limited especially due to the small patient population (2 × 19 patients), a firm conclusion about the efficacy of CS cannot be made. Moreover, weighted methods might also be a useful approach to help compare groups more effectively. Furthermore, there were different causes leading to the cytokine storm in our study population. A precise evaluation of the cause of the cytokine storm or the applying of the CRS criteria was not performed. It was beyond the scope of this study to evaluate whether CS therapy might be beneficial in specific disease patterns. Finally, the initiation of CS treatment was at the discretion of the attending physician. A bias regarding potential confounders that led to CS therapy might not have been detected with our matching methods. Therefore, it cannot completely be excluded.

## Conclusion

There was no difference in the reduction in IL-6, hemodynamic stabilization, or in-hospital mortality in patients treated with Cytosorb^®^ compared to a matched patient population. Its use should therefore be questioned in patients with cytokine storm. However, differences might not have been detected despite propensity score matching, especially with regard to specific patient populations.

## Data Availability

All data generated during this study are included in this article.

## Abbreviations

AKI: Acute kidney injury
BMI: Body mass index
CRP: C-reactive protein
CS: Cytosorb^®^
ECMO: Extracorporeal membrane oxygenation
ICU: Intensive care unit
IL-6: Interleukin-6
PS: Propensity score
KRT: Kidney replacement therapy
SAPS: Simplified Acute Physiology Score
